# Genomic and Epigenomic Landscape of Juvenile Myelomonocytic Leukemia

**DOI:** 10.3390/cancers14051335

**Published:** 2022-03-04

**Authors:** Claudia Fiñana, Noel Gómez-Molina, Sandra Alonso-Moreno, Laura Belver

**Affiliations:** 1 Cancer and Leukemia Epigenetics and Biology Program, Josep Carreras Leukemia Research Institute (IJC), 08916 Badalona, Barcelona, Spain; cfinana@carrerasresearch.org (C.F.); ngomez@carrerasresearch.org (N.G.-M.); salonso@carrerasresearch.org (S.A.-M.); 2 Immuno Procure, Catalan Institute of Oncology (ICO), 08916 Badalona, Barcelona, Spain

**Keywords:** juvenile myelomonocytic leukemia, RAS pathway, DNA methylation, experimental therapeutics

## Abstract

**Simple Summary:**

Juvenile myelomonocytic leukemia (JMML) is a rare pediatric myelodysplastic/myeloproliferative neoplasm characterized by the constitutive activation of the RAS pathway. In spite of the recent progresses in the molecular characterization of JMML, this disease is still a clinical challenge due to its heterogeneity, difficult diagnosis, poor prognosis, and the lack of curative treatment options other than hematopoietic stem cell transplantation (HSCT). In this review, we will provide a detailed overview of the genetic and epigenetic alterations occurring in JMML, and discuss their clinical relevance in terms of disease prognosis and risk of relapse after HSCT. We will also present the most recent advances on novel preclinical and clinical therapeutic approaches directed against JMML molecular targets. Finally, we will outline future research perspectives to further explore the oncogenic mechanism driving JMML leukemogenesis and progression, with special attention to the application of single-cell next-generation sequencing technologies.

**Abstract:**

Juvenile myelomonocytic leukemia (JMML) is a rare myelodysplastic/myeloproliferative neoplasm of early childhood. Most of JMML patients experience an aggressive clinical course of the disease and require hematopoietic stem cell transplantation, which is currently the only curative treatment. JMML is characterized by RAS signaling hyperactivation, which is mainly driven by mutations in one of five genes of the RAS pathway, including *PTPN11*, *KRAS*, *NRAS*, *NF1,* and *CBL*. These driving mutations define different disease subtypes with specific clinico-biological features. Secondary mutations affecting other genes inside and outside the RAS pathway contribute to JMML pathogenesis and are associated with a poorer prognosis. In addition to these genetic alterations, JMML commonly presents aberrant epigenetic profiles that strongly correlate with the clinical outcome of the patients. This observation led to the recent publication of an international JMML stratification consensus, which defines three JMML clinical groups based on DNA methylation status. Although the characterization of the genomic and epigenomic landscapes in JMML has significantly contributed to better understand the molecular mechanisms driving the disease, our knowledge on JMML origin, cell identity, and intratumor and interpatient heterogeneity is still scarce. The application of new single-cell sequencing technologies will be critical to address these questions in the future.

## 1. Introduction

Juvenile myelomonocytic leukemia (JMML) is a rare and very heterogeneous myelodysplastic/myeloproliferative neoplasm of early childhood resulting from the malignant transformation of hematopoietic stem/progenitor cells (HSPCs) and characterized by the hyperactivation of the RAS signaling pathway. Children with JMML typically show symptoms related to the infiltration of the bone marrow (BM) and other organs by malignant mature and immature myeloid cells. Formal diagnosis of JMML requires the presence of prominent monocytosis (≥1 × 10^9^/L), a low proportion of blasts in the BM (<20%), splenomegaly, absence of BCR-ABL fusions, and mutations in genes encoding for proteins of the RAS signaling pathway [1]. Granulocyte–macrophage colony-stimulating factor (GM-CSF) in vitro hypersensitivity is a common hallmark in JMML and can be used as a diagnostic criterion in patients in which RAS pathway mutations are not identified [2,3]. Although JMML karyotype is predominantly normal, recurrent cases of monosomy 7 are observed in approximately 25% of the patients, as well as other karyotype abnormalities involving 10% of cases [4]. JMML therapeutic options are scarce, with early allogeneic hematopoietic stem cell transplantation (HSCT) being the only effective therapy for achieving long-term disease control. However, this treatment entails a significant risk of transplant-related mortality and the overall survival at five years in treated patients remains at 64%, largely due to unsuccessful HSCT [3,5,6]. 

Despite the major advances in the study of the underlying molecular defects in JMML, this disease is still a puzzling disorder with a wide variety of phenotypes and outcomes, ranging from rare self-limiting forms that spontaneously resolve, to aggressive cases prone to relapse and with dismal prognosis. In this context, the characterization of the genomic and epigenomic landscapes in JMML has not only contributed to identify novel oncogenic mechanisms involved in the pathogenesis of the disease, but also provided critical insights in predicting patient prognosis and making clinical decisions.

## 2. Genetic Alterations in JMML

**Figure 1:**
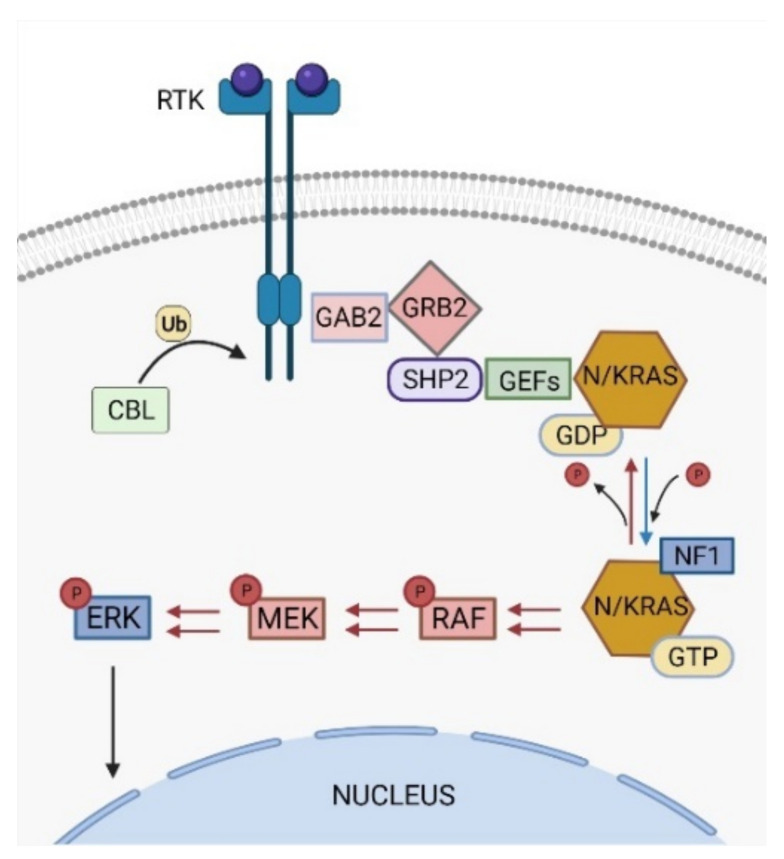
The RAS signaling pathway. NRAS and KRAS are small GTPase switch proteins that act downstream receptor and non-receptor tyrosine kinases (RTKs and TKs). RAS activation status is regulated by a two-stage molecular system directed by phosphorylation and dephosphorylation changes in RAS, which are regulated by the opposing activities of guanine nucleotide-exchange factors (GEFs) and GTPase activating proteins (GAPs). RTK/TK stimulation promotes the recruitment of adaptor proteins (such as GAB2, GRB2, and SHP2) and GEFs to mediate RAS-GDP phosphorylation to a RAS-GTP active status. Active RAS then triggers a signaling cascade that sequentially activates RAF, and phosphorylates MEK and ERK proteins, which ultimately signal to the nucleus to control specific cell functions such as proliferation, survival, and differentiation, among others. RAS pathway is inactivated by the activity of GAPs, such as NF1, which promote RAS-GTP dephosphorylation to a RAS-GDP inactive form. In addition, the ubiquitin ligase CBL can also act as a negative regulator of the RAS pathway by targeting active RTKs for proteasomal degradation. Figure was created with BioRender.com.

The RAS signaling pathway is one of the most studied pathways in cell biology and its deregulation is widely observed in approximately 40% of cancer patients [7]. Under normal conditions, RAS pathway activation triggers a phosphorylation signaling cascade that ultimately boosts cell proliferation, survival, differentiation, and migration, among other functions (Figure 1) [8]. 

Around 90% of JMML patients carry mutations in one of five genes of the RAS pathway, including *PTPN11, NRAS, KRAS, CBL,* and *NF1*. Concomitantly, some other secondary mutations affecting either additional RAS pathway components or external elements have been described in JMML patients [9].

### 2.1. PTPN11

**Figure 2:**
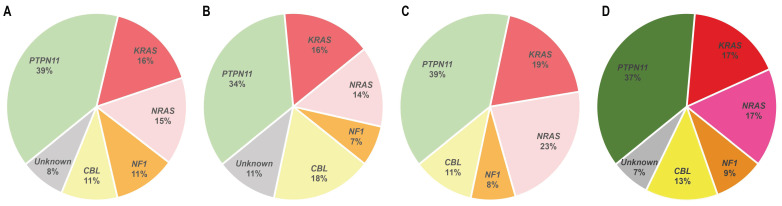
Distribution of RAS pathway mutations in children with JMML. Data reported by Lipka et al. [25] (**A**), Murakami et al. [37] (**B**), and Caye et al. [29] (**C**). Panel (**D**) summarizes the three studies. NS cases were excluded from the analysis.

Activating *PTPN11* somatic mutations are the most common genetic drivers of JMML, accounting for approximately 35–40% of the patients (Figure 2), and are associated with an aggressive clinical course and poor disease outcome [10,11,12]. The *PTPN11* gene encodes for the protein tyrosine phosphatase SHP2, which acts downstream of various receptor and cytoplasmic tyrosine kinases, and promotes RAS signaling activation (Figure 1) [13].

SHP2 structure consists in two tandem Src homology 2 recognition domains (N-SH2 and C-SH2), followed by a catalytic protein tyrosine phosphatase (PTP) domain, and a C-terminal hydrophilic tail containing phosphorylation sites [14]. In the inactive state, the N-SH2 domain engages the PTP domain, keeping the phosphatase in a close autoinhibited conformation [15]. Under physiological conditions, the binding of tyrosine-phosphorylated ligands to the tandem SH2 domains stabilizes an open SHP2 conformation that renders the active site accessible and allows the dephosphorylation of target substrates [16]. 

JMML *PTPN11* mutations occur mainly in the N-SH2 domain of SHP2, particularly in the residues G60, D61, A72, and E76, which account for more than 70% of *PTPN11*-mutated patients [12]. These mutations result in ligand-independent forms of the enzyme that constitutively activate downstream effectors of the RAS pathway [17].

Interestingly, germline mutations in *PTPN11* are highly prevalent in Noonan Syndrome (NS), a developmental disorder characterized by unusual facial features, a restricted growth, and cardiovascular defects [18]. Approximately 5% of NS patients are affected by a mild myeloproliferative disorder, which is hematologically indistinguishable from JMML, but usually resolves spontaneously without intervention [12,19]. However, a small subset of NS patients (approximately 3%) progress into bona fide JMML and half of them die within the first month of life [19]. The distribution of NS-associated *PTPN11* germline mutations differs from the one observed in *PTPN11*-mutated JMML patients and results in weaker SHP2 forms [12,19]. 

### 2.2. NRAS and KRAS

Approximately 25–30% of JMML patients present heterozygous somatic-activating mutations in the *RAS* paralogs *NRAS* and *KRAS* (Figure 2) [20]. The NRAS and KRAS proteins are small GTPases that act as binary molecular switches of the RAS signaling pathway. NRAS and KRAS are active when bound to guanosine triphosphate (GTP) and inactive when bound to guanosine diphosphate (GDP) [20]. This phosphorylation exchange is regulated by the opposing activity of guanine nucleotide-exchange factors (GEFs) and GTPase-activating proteins (GAPs) (Figure 1) and results in allosteric conformational changes at the RAS protein G domain, which is critical for RAS activation [21].

JMML mutations occur mostly in the residues G12, G13, and Q61, which are located at the G domain both in KRAS and NRAS, and render the proteins insensitive to GAP inactivation, stabilize their GTP-bound conformation, and/or affect nucleotide-exchange rate [22,23,24,25,26]. Duplication of *NRAS* and *KRAS* oncogenic alleles through acquired uniparental disomy (UPD) by mitotic recombination is observed in some JMML patients and is associated with higher aggressiveness and worst outcomes [27,28]. Interestingly, there is a strong association between *KRAS* mutations and monosomy 7, being the latter present in approximately 50% of *KRAS*-mutated JMML patients [29]. This observation suggests an interaction between the oncogenic mechanisms driven by these two genetic alterations. 

Germline mutations in RAS proteins have been described in different RASopathies, including NS. However, the distribution of these mutations is different to the one observed in JMML patients harboring *NRAS*/*KRAS* somatic mutations, and the evidence pointing to a driver role in JMML leukemogenesis is scarce [30,31,32,33]. A *NRAS* germline mutation at G13D was found as a possible driver event in a JMML patient, and somatic mosaicism of *NRAS* mutations, acquired at early developmental stages, has been reported in two JMML patients, who developed a mild clinical form of the disease [34,35]. In addition, a *KRAS* germline mutation at T58I was identified in a NS patient who presented a JMML-like disorder [36]. 

### 2.3. CBL

Germline and somatic *CBL* loss-of-function mutations account for 10–15% of JMML patients [38,39]. Germline *CBL* mutations are associated to Noonan-like CBL syndrome, a constitutional disease that is presented as a mild form of NS with a heterogeneous set of clinical features with a variable penetrance (developmental delay, reduced growth, facial dysmorphism, among others) and an increased risk to develop JMML [38,40,41]. Disease progression to JMML normally occurs after loss of heterozygosity of the *CBL* wild type allele, typically through UPD encompassing the *CBL* locus [42,43,44]. *CBL*-mutant JMML patients usually develop indolent forms of the disease that resolve spontaneously, and only a subset of them require HSCT treatment [38,45].

The *CBL* gene encodes for the RING E3 ubiquitin ligase CBL that acts as negative regulator of activated protein tyrosine kinases by promoting their targeting for degradation by the proteasome (Figure 1) [46]. The CBL protein comprises a highly conserved N-terminal tyrosine kinase-binding (TKB) domain, followed by a central region containing a helical linker and a RING finger domain, which are critical for CBL ubiquitin ligase activity, and a C-terminal proline-rich sequence, that mediates interactions of CBL with SH3 domain-containing proteins [46,47].

Most JMML somatic mutations in CBL occur in the linker region, especially in Y371, or in different residues at the RING finger domain, and lead to the loss of E3 ubiquitin ligase activity against tyrosine kinase substrates [9,38,42]. CBL linker mutants have been shown to enhance LYN- and JAK2-mediated GM-CSF signaling by activating the PI3K/AKT/mTOR and JAK/STAT pathways, respectively [48,49]. Moreover, CBL can also regulate the RAS pathway through an indirect mechanism involving the adaptor protein GRB2 [50]. CBL interaction with GRB2 prevents the binding of this protein to SOS, a GEF that promotes the formation of active RAS-GTP complexes (Figure 1) [51]. *CBL* loss-of-function mutations impair the binding of CBL to GRB2, indirectly promoting RAS pathway activation by allowing GRB2-SOS interaction and SOS-mediated GDP/GTP nucleotide exchange in RAS proteins [52,53]. Interestingly, GRB2 is a core component of a multiprotein complex that includes SHP2, among other factors (Figure 1) [13,54]. The interaction of this complex with SOS allows SHP2 to dephosphorylate the RAS residue Y32, which increases the binding of RAS to RAF, thus contributing to RAS activation [55]. This adaptor activity of SHP2 and the inhibitory role of CBL in the regulation of GRB2-SOS complex function illustrate how *CBL* loss-of-function mutations and *PTPN11* gain-of-function mutations (through the adaptor activity of SHP2) functionally converge through a similar molecular mechanism to induce RAS activation, which could explain why these two genetic alterations are mutually exclusive in JMML [42].

### 2.4. NF1

Germline and somatic loss-of-function mutations at the *NF1* tumor suppressor gene are found in 10% to 15% of JMML patients (Figure 2) [4]. Germline *NF1* mutations are associated to neurofibromatosis type 1, a common autosomal congenital disorder characterized by the presence of café-au-lait macules, skinfold freckling, development of tumors of the nervous system, and overlapping features with other RASopathies, such as NS and Legius syndrome [56]. Although it is not a common complication, *NF1*-mutated neurofibromatosis type 1 patients have an increased predisposition to develop JMML, with a 200- to 350-fold increased risk compared to their wild type *NF1* counterparts [4]. In *NF1*-mutant patients, JMML progression is triggered by loss of heterozygosity of the wild type *NF1* allele, typically by UPD or compound-heterozygous mutations [57].

*NF1* encodes for neurofibromin, a GAP that functions as negative regulator of the RAS signaling pathway. Neurofibromin binds to RAS family proteins and stimulates the hydrolysis of active RAS-GTP to RAS-GDP inactive forms (Figure 1) [58,59,60]. 

Most of JMML reported alterations in *NF1* are nonsense or frameshift mutations resulting in a truncated protein due to a premature termination codon [61,62]. *NF1* loss-of-function mutations result in a reduced dephosphorylation of RAS-GTP activated proteins and confer sustained activation of the RAS signaling pathway [63].

### 2.5. Other Driver Genetic Alterations in the RAS Pathway

Although *PTPN11*, *NRAS*, *KRAS*, *CBL,* and *NF1* mutations account for approximately 90% of JMML cases, around 10% of the patients that are clinically diagnosed with JMML do not present mutations in any of these five RAS pathway genes [37]. However, several studies have reported other genetic alterations that can possibly act as molecular drivers of the disease in cases of unknown origin.

Gain-of-function somatic mutations affecting known oncogenic hotspots of the *RAS* genes were identified in *RRAS* (Q87L) and *RRAS2* (Q72L) in two independent patients that lacked any of the canonical JMML mutations at diagnosis, supporting a driver role of these alterations [64]. In addition, the analysis of a cohort of 16 patients presenting a JMML-like phenotype without mutations in any of the five canonical JMML genes revealed three patients that harbored gain-of-function *ALK* and *ROS1* tyrosine kinase fusions, including *RANBP2-ALK*, *DCTN1-ALK,* and *TBL1XR1-ROS1* [37]. Fusions involving the tyrosine kinase genes *PDGFRB* (*SPECC1*-*PDGFRB* and *NDEL1-PDGFRB*) and *FLT3* (*CCDC88C*-*FLT3*) were also identified in case reports of JMML-diagnosed patients lacking mutations in the classical JMML drivers [65,66,67]. Similar rearrangements involving these tyrosine kinases have been also described in other hematologic malignancies [68,69,70,71,72], supporting the role for these alterations as an alternative oncogenic mechanism of RAS pathway hyperactivation in leukemia transformation. However, although these patients harboring tyrosine kinase fusions recapitulate the clinical features of JMML, it is still a matter of controversy whether they should be diagnosed as JMML or instead represent an as yet undefined category of myelodysplastic/myeloproliferative neoplasms. Further research must be carried out to shed light on this debate.

### 2.6. Secondary Genetic Alterations in JMML

JMML is characterized by a low mutational rate, suggesting that a limited number of genetic alterations is required to support JMML leukemogenesis [29]. However, secondary mutational events that contribute to the pathogenesis of the disease have been recurrently identified in different cohorts of JMML patients.

**Figure 3:**
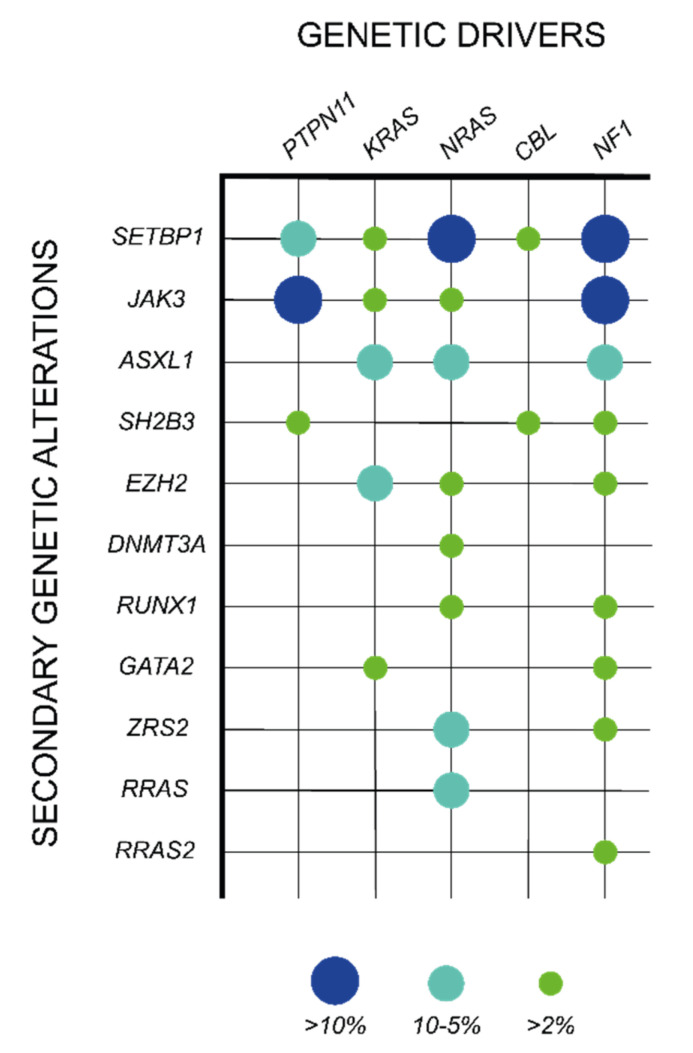
Association of secondary mutations with specific genetic drivers in JMML. Data reported by Murakami et al. [37], Caye et al. [29], and Stieglitz et al. [64,85]. Colored circles represent the relative frequency of a secondary mutation in patients with each of the five JMML canonical drivers (green > 2%; light blue 5–10%; dark blue > 10%).

Although the mutations in the five canonical JMML genes are in general mutually exclusive, around 10% of JMML patients harbor co-existing alterations in these genes, being the association of *PTPN11* and *NF1* mutations the most common co-mutational event [37]. In addition, approximately 50% of the cases present secondary somatic mutations in other genes, which are specifically associated with particular RAS pathway initiating lesions and expand clonally, indicating a cooperative role with the driver event in JMML maintenance (Table 1; Figure 3) [29]. Within the RAS pathway, heterozygous mutations in signaling components (*RRAS*, *RAC2,* and *SOS1*) or RAS regulators (*PLXNB2*, *ABI1,* and *PDE8A*) have been described in JMML in combination with some of the classical driver events and contribute to JMML pathogenesis by enhancing RAS pathway activation (Table 1) [29,37,64]. In addition, other genetic events outside the RAS pathway have been reported as major secondary mutations in some JMML subsets. Among them, *SETBP1* activating mutations are the most prevalent genetic events, being present in around 30% of JMML patients and correlating with poorer disease outcomes [73,74]. SETBP1 directly binds to SET, which functions as an inhibitor of the protein phosphatase PP2A, a well-known tumor suppressor in hematopoietic malignancies [75,76]. This interaction protects SET from degradation, potentiating its inhibitory activity over PP2A [77]. *SETBP1* mutations disrupt the degron motif of the protein, resulting in an impaired proteasome cleavage and subsequent SETBP1 protein accumulation, which further enhances the inhibitory effects of SET over PP2A and support leukemia cell proliferation [77,78]. In addition to this function, SETBP1 has been shown to directly bind AT-rich promoter regions and contribute to the transcriptional activation a set of target genes that include the hematopoietic master regulators *HOXA9* and *HOXA10* [79,80]. This effect correlates with an increase in myeloid progenitor self-renewal capacity in Setbp1-overexpressing mouse bone marrow, supporting the existence of additional PP2A-independent oncogenic mechanisms driven by SETBP1 aberrant expression [79]. In JMML, *SETBP1* mutations associate with *PTPN11* or *NRAS* somatic mutations [74]. Interestingly, a recently published mouse model combining *SETBP1* and *NRAS* mutations has shed some light on the mechanism of interaction between these two factors by showing that the aberrant expression of SETBP1 enhances both NRAS gene expression signature and NRAS-driven MAPK protein phosphorylation [81].

Along with the mutations in *SETBP1*, other secondary genetic alterations are also observed as clonal events in JMML patients, including mutations in hematopoietic commitment transcription factors (*RUNX1*, *GATA2, RARA,* and *HOXA11*), spliceosome components (*ZRSR2*), cAMP pathway components (*PDE8A*), structural protein components and regulators (*WASP, DYNC1H1, TNS3, COL22A1, KRT1,* and *SMC1A*), or JAK/STAT pathway components (*JAK3* and *SH2B3*), among others (Table 1) [29,37,64,73,74,81,84,86,87,88,89]. These secondary mutations are associated with an aggressive clinical course of the disease and an increased risk of relapse after HSCT [64].

Of special relevance are the genetic alterations that affect components of the epigenetic machinery, which are observed in approximately 15% of JMML patients [29,64]. These genetic alterations include mostly mutations at the genes encoding for the polycomb repressive complex 2 (PRC2) core component EZH2 and the PRC2-associated factor ASXL1. Of note, the *EZH2* gene is located at chromosome 7 and all JMML-associated *EZH2* mutations are hemizygous due to co-occurring monosomy 7 [29,64]. In addition to these mutations, copy number variations (CVN) in genes encoding for other PRC2 complex components and associated factors, such as *SUZ12, AEBP2, CDYL,* or *JARID*, have been found in some subsets of JMML patients [29]. Mutations at the *DNMT3A* gene, encoding for the DNA methyltransferase 3 alpha, have been also described; however, the recurrence of this genetic alterations in JMML is lower than in other hematologic malignancies [64,90].

The PRC2 complex directs the transcriptional repression of target genes by catalyzing histone H3 trimethylation at lysine 27 (H3K27me3) [91]. Interestingly, a recent study demonstrated that JMML patients that present an impaired PRC2 activity show a global decrease in H3K27me3 and a concomitant increase in H3K27 acetylation, suggesting a critical role of PRC2-associated mutations in regulating the JMML transcriptional program at the epigenetic level [29]. These findings illustrate the crosstalk between genetics and epigenetics in JMML and highlight the importance of a better understanding of the oncogenic mechanisms driven by epigenetic dysregulation during JMML leukemogenesis.

## 3. Epigenetic Alterations in JMML

Although genetic mutational events are generally considered the main drivers of cancer transformation, alterations in the epigenetic landscape of tumor cells have a critical role in cancer pathogenesis, providing additional mechanisms to consolidate specific oncogenic transcriptional programs [92]. Over the last decade, several research groups have explored the epigenetic landscape in JMML and identified important alterations in the methylome of JMML cells that correlate with the severity and prognosis of the disease. This fact highlights the urgent need of a better understanding of the oncogenic mechanisms driven by JMML epigenetic aberrations and postulates the use of epigenetic modifiers as a potential therapeutic strategy for the treatment of this disease.

### 3.1. Early Studies on DNA Methylation in JMML

**Figure 4:**
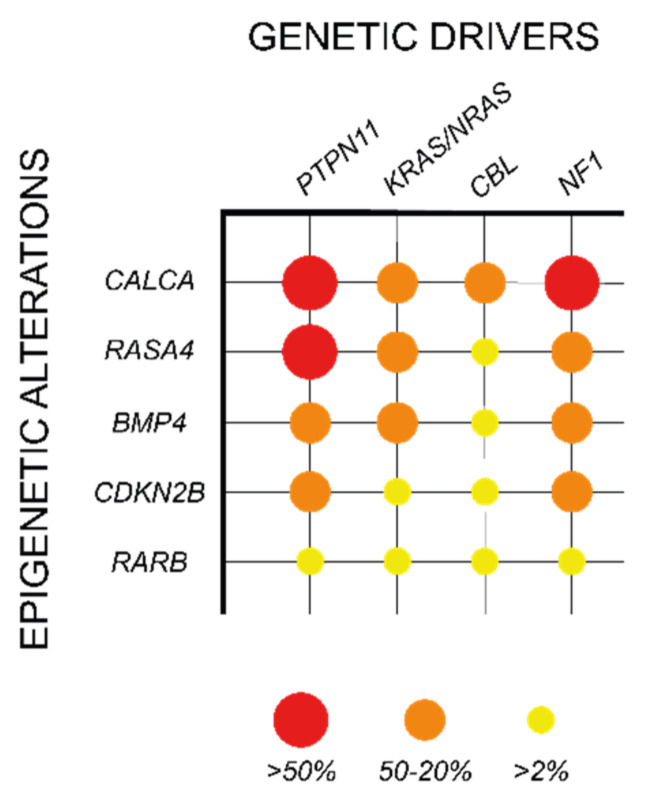
Association of epigenetic alterations with specific genetic drivers in JMML. Data reported by Olk-Batz et al. [94] and Poetsch et al. [96]. Colored circles represent the relative frequency of a specific epigenetic alteration in patients with each of the five JMML canonical drivers (yellow > 2%; orange 20–50%; red > 50%).

CpG island methylation at gene promoters is an important repressive mechanism of gene expression, which has been shown to play a relevant oncogenic role in different cancers, including myeloid malignancies [93]. In JMML, methylation in CpG islands was first analyzed in a European cohort of 86 patients, in which 14 candidate genes were selected based on their hypermethylation status in other cancer types (*CALCA*, *CDKN1C*, *CDKN2B*, *DAPK1*, *MGMT*, *MLH1*, *RARB*, *RASSF1*, *SOCS1*, and *TP73*) or their involvement in RAS signaling (*BMP4*, *PAWR*, *RASA1*, and *RECK*). Among the selected candidates, four genes were found recurrently hypermethylated, including *BMP4*, *CALCA*, *CDKN2B*, and *RARB* (Table 2; Figure 4). Interestingly, this hypermethylation phenotype correlated with poorer prognosis and a high risk of treatment failure due to relapse after HSCT [94].

These results were further validated in a Japanese cohort of 92 JMML patients, in which the CpG methylation status of 16 genes was analyzed, including nine genes that were common to the European study (*CALCA*, *CDKN2B*, *DAPK1*, *MGMT*, *MLH1*, *RARB*, *RASSF1*, *TP73,* and *BMP4*) and seven new candidate genes (*APC*, *CDH13*, *CDKN1A*, *CHFR*, *ESR1*, *H19*, and *IGF2AS*). This study not only confirmed the hypermethylation of *BMP4*, *CALCA*, *CDKN2B*, and *RARB*, but also provided a novel prognostic tool based on the “aberrant methylation score” (AMS). AMS stratifies the patients in three groups based on the number of hypermethylated genes (0, 1–2, or 3–4) and predicts their 5-year overall survival and transplant-free survival, with high AMS patients presenting a dismal prognosis [95].

In addition to the genes identified in these studies, hypermethylation in other genes such as *RASA4*, *CREBBP*, and *AKAP12* was also observed in JMML patients in correlation with a poor survival and high risk of relapse after HSCT, further supporting the significance of DNA methylation in aggressive JMML phenotypes (Table 2; Figure 4) [96,97,98].

### 3.2. The JMML Methylation Landscape

Early methylation studies in JMML focused on the analysis of specific gene subsets. However, a global view on the DNA methylation landscape in JMML was not obtained until the first genome-wide CpG methylation analyses were performed [25,37,99]. Based on these studies, patients were clustered according to their global methylation status in three groups: low methylation (LM), intermediate methylation (IM), and high methylation (HM). These groups were not only associated with particular outcomes [99], but also to specific molecular profiles [25,37]. Thus, LM patients showed enrichment of somatic *NRAS* and *CBL* mutations and presented high survival rates, the IM group was associated to somatic *KRAS* mutations and monosomy 7, and the HM patients showed an enrichment in *PTPN11* mutations and were characterized by a poor clinical outcome [25]. Interestingly, the analysis of HM samples showed an upregulation in the expression of the genes encoding for the DNA methyltransferases *DNMT1* and *DNMT3B*, suggesting an association between activation of DNA methylation mechanisms and specific JMML mutational profiles [25]. Overall, these data further supported the diagnostic value of DNA methylation in JMML patients.

### 3.3. DNA Methylation as a Prognostic Tool in JMML

The accumulating evidences on the role of DNA methylation in the pathogenesis of JMML has led to the recent publication of an international JMML stratification consensus, which has defined the parameters and characteristics of the different DNA methylation subgroups in JMML [100]. In here, the Illumina Infinium 450 k/EPIC array technology was applied to develop and validate a machine learning classifier for prospective patient classification. 

To complement this novel prognostic tool, a new technique called Digital Restriction Enzyme Analysis of Methylation (DREAM) was developed to provide an easy and robust method to evaluate DNA methylation in JMML clinical samples [101]. In this method, DNA is sequentially digested with a methylation-sensitive restriction enzyme (SmaI), followed by a restriction enzyme that is tolerant to DNA methylation (XmaI). This digestion results in the generation of two DNA fragment types, carrying either a CCGGG tag in their 5′ end in methylated DNA sites, or a GGG tag in unmethylated DNA sites. These fragments are then analyzed by Next-Generation Sequencing (NGS), allowing a quantitative whole-genome evaluation of DNA methylation [102]. DREAM represents a promising and cost-efficient technology for the evaluation of DNA methylation in clinical settings; however, further research will be required to address its implementation and determine the robustness of the approach in different laboratories. 

The standardized use of DNA methylation as a biomarker in JMML and the incorporation of techniques such as Illumina Infinium 450 k/EPIC arrays or new techniques such as DREAM as prognostic methods represent a unique breakthrough for the stratification and management of JMML patients. These tools will not only improve clinical decision-making, but also contribute to optimize the inclusion criteria of specific JMML subsets in future clinical trials.

## 4. Genetic and Epigenetic Therapeutic Targets for the Treatment of JMML

JMML has historically represented a clinical challenge mainly due to the limited number of therapeutic options for its treatment and the inevitable fatal outcome in children with the most aggressive forms of the disease [103,104]. HSCT is currently the only effective therapy for achieving long-term disease control in JMML [103]. However, the advances in the characterization of the molecular mechanisms driving and supporting the progression of JMML have provided new potential genetic and epigenetic therapeutic targets that are currently being explored in different preclinical assays and clinical trials as alternative treatments for JMML (Table 3). These studies hold promise for an improvement in the pre- and post-HSCT management of the disease, and might have a direct impact on the prognosis and survival of JMML patients in the future.

### 4.1. JMML Therapeutic Targets on Signaling Pathways

#### 4.1.1. RAS Pathway Targeting

Although RAS hyperactivation is a hallmark in JMML, the therapeutic targeting of factors involved in RAS signaling has been shown to provide limited benefits due to the frequent treatment-associated toxicities, and the functional redundancy and complexity of the pathway [105,106,107]. However, some studies have explored the use of different tyrosine kinase (TK) and mitogen-activated protein kinase (MEK) inhibitors to target RAS signaling, as a therapeutic alternative for JMML treatment.

A small subset of clinically-diagnosed JMML patients harbor fusions involving diverse RAS components, which result in the abnormal activation of specific TKs, such as *ALK*, *ROS1*, or *FLT3* [37,67]. Some examples illustrate how drug repurposing of TK inhibitors (TKIs) that are approved for the treatment of other pathologies could have a potential therapeutic benefit on this atypical subset of JMML patients. Such is the case of crizotinib, a potent inhibitor of ALK and ROS1 that is approved for the treatment of non-small cell lung cancer [108]. Current evidences on the benefits of crizotinib treatment on JMML are limited to the case of a patient carrying *RANBP2-ALK* fusion that was refractory to conventional cytotoxic chemotherapy. The addition of crizotinib to the treatment resulted in complete molecular remission and allowed successful HSCT in this patient [37]. In the same study, another two patients who harbored *ALK* or *ROS1* fusions were not treated with crizotinib and succumbed due to tumor progression [37].

Similar to this case, the use of another TKI, sorafenib, has been also explored in the context of JMML [67]. Sorafenib, is currently approved for the treatment of different types of cancer, including acute myeloid leukemia patients that carry an activating internal tandem duplication mutation on FLT3 (FLT3-ITD) [109]. A JMML patient harboring a *CCDC88C-FLT3* fusion who was refractory to conventional chemotherapy, was treated with sorafenib, resulting in FLT3 inhibition and cytogenetic remission, which also in this case allowed successful HSCT [67].

In the case of *PTPN11*-mutant JMML, a recent study has identified the non-receptor tyrosine kinase ACK1 (encoded by the *TNK2* gene) as a potential therapeutic target for these patients [110]. In vitro assays showed that the ACK1-specific inhibitors AIM-100 and XMD8-87 can reduce the transforming potential of JMML-associated *PTPN11* mutations [110]. Clinically, the use of dasatinib, a TKI that is approved for the treatment of different types of leukemia and can target ACK1 (among other TKs), was shown to reduce disease burden and provide extended survival in a *PTPN11*-mutant JMML patient [110].

Together, these data postulate the use of TKIs as a potential therapeutic approach to achieve disease remission and facilitate HSCT in some JMML patient subsets; however, further research is required to validate these observations.

In addition to TKIs, the use of MEK inhibitors (MEKi) in JMML has also been explored. Especially remarkable is the case of trametinib, a MAP2K1/MAP2K2 inhibitor that is currently approved for the treatment of metastatic melanoma [111]. The first evidences on the potential therapeutic effect of trametinib in JMML were obtained in in vitro assays using induced pluripotent stem cells (iPSCs) derived from *PTPN11*- and *CBL*-mutant JMML cells [112]. In this experimental setting, trametinib treatment resulted in efficient RAS signaling and cell growth inhibition in *PTPN11*-mutant iPSCs [112]. More recently, trametinib has also been shown to provide a survival benefit in a mouse model in which leukemogenesis is driven by the combined expression of JMML-associated *NRAS* and *SETBP1* mutations [81]. The application of trametinib for the treatment of JMML is currently being explored in a phase II clinical trial, which aims to determine the safety and efficacy of this drug in refractory or relapsed JMML patients (ClinicalTrials.gov Identifier: NCT03190915).

#### 4.1.2. Targeting of Other Signaling Pathways

The PI3K/AKT/mTOR pathway is one of the main downstream effectors of the RAS pathway and it is also affected by RAS hyperactivation [106]. In JMML, some studies have provided evidence of the beneficial effects of PI3K/AKT/mTOR inhibition. The use of rapamycin, a specific and potent mTOR inhibitor, has been shown to reduce signaling and proliferation of JMML-derived *PTPN11*-mutant iPSCs in vitro [112]. Similar results were obtained in the same experimental setting upon treatment with idelalisib, a PI3Kδ inhibitor [112]. Moreover, the use of idelalisib on primary JMML cells resulted in a dose-dependent reduction in GM-CSF hypersensitivity in two of the three samples analyzed [113]. Finally, both rapamycin and idelalisib have also shown a therapeutic effect in in vivo leukemia mouse models driven by the expression of JMML-associated *PTPN11* mutations [114,115].

Secondary mutations affecting components of the JAK/STAT pathway, such as *JAK3* or *SH2B3*, are recurrently found in JMML patients, indicating a relevant role of this signaling pathway in the pathogenesis of JMML [29,64,73,74]. Therefore, targeting the JAK/STAT pathway could potentially report beneficial effects in some JMML patient subsets. In this context, a recent in vitro study revealed that JMML-derived *CBL*-mutant iPSCs are sensitive to the JAK inhibitors momelotinib and ruxolitinib, reducing both cell proliferation and aberrant signaling [112].

Although clinical data on the use of these inhibitors in JMML are not available, the promising results obtained in in vitro and in vivo preclinical models support a potential therapeutic benefit of these treatments in JMML patients. However, further research will be required to explore this possibility in a clinical setting.

### 4.2. Epigenetic Therapeutic Targets in JMML

DNA hypermethylation has been showed to be a hallmark in the most aggressive cases of JMML [25,37,99]. For that reason, the use of the DNA methyltransferase inhibitor azacitidine has been explored as a potential therapeutic agent for the treatment of this disease, alone or in combination with conventional cytotoxic chemotherapy [116]. Azacitidine use in JMML was first reported on a patient that achieved complete clinical and genetic remission of the disease after eight cycles of treatment prior to HSCT [117]. Later on, a retrospective study showed that low-dose azacitidine was effective and tolerable in JMML, and documented another three JMML cases in which this drug induced complete remission before HSCT [118]. Finally, the beneficial effects of pre-HSCT azacitidine treatment were formally validated in a phase II clinical trial (NCT02447666) that demonstrated that the use of this drug as a single agent is a suitable option for newly diagnosed JMML patients, independent of their methylation status [116]. In addition, the histone deacetylase inhibitor vorinostat, is currently being explored in a phase I clinical trial (NCT03843528) in combination with low dose azacitidine for the treatment of pediatric myeloid malignancies, including JMML. Further clinical studies will be required to determine long-term safety and efficacy of these treatments in JMML.

**Table 3:** Experimental therapeutic strategies for JMML treatment.

	Pathway	Target	Inhibitor	Status	References
Signaling pathway inhibitors	RAS	ACK1	Dasatinib	In vitro	[110]
		ALK/ROS1/MET	Crizotinib	In vitro	[37]
		MEK	Trametinib	Phase II clinical trial	[81,112,119,120]
		FLT3	Sorafenib	Clinical use	[67]
	PI3K	mTOR	Rapamycin	Preclinical in vivo	[112,114,115]
		PI3Kδ	Idelalisib	Preclinical in vivo	[112,113,114,115]
	JAK/STAT	JAK1/JAK2	Momelotinib	In vitro	[112]
		JAK1/JAK2	Ruxolitinib	In vitro	[112]
Epigenetic inhibitors	Methylation	DNMTs	Azacitidine	Clinical use	[37,116,117,118,121,122]
	Acetylation	HDACs	Vorinostat	Phase I clinical trial	

## 5. Current Challenges and Future Perspectives in JMML Research

Despite of the advances in the genetic and epigenetic characterization of JMML, there are still several experimental challenges and clinically relevant open questions that remain to be addressed.

### 5.1. JMML Experimental Models

JMML research has been traditionally hindered due to its low incidence (1.2 cases per million children under 14 years of age), and the impossibility of maintaining primary JMML cells in vitro for extended periods of time or establishing immortalized JMML cells lines [123,124]. In this context, the development of methods to generate JMML patient-derived xenografts (PDX) and iPSCs has been instrumental to overcome low sample number difficulties and provide an unlimited source of JMML cells for experimental purposes.

JMML PDXs have been established in immunodeficient NSG (NOD/SCID/IL2rγ^−/−^) and NSG-S (NOD/SCID/IL2rγ^−/−^/IL-3/GM/SF) mice, and in both cases these models present not only the immunophenotypical features of the primary sample, but also maintain the clonal diversity of the original tumor [84,125]. On the other hand, different reprogramming techniques have been successfully applied towards the generation of JMML-derived iPSCs, providing a unique experimental tool to perform high-throughput analysis, such as drug or CRISPR screens [112,126,127,128]. However, the lack of a physiological bone marrow microenvironment in iPSC models gives rise to important concerns regarding whether these systems can faithfully recapitulate the molecular and functional features of the tumor. Future JMML modeling efforts should focus on the establishment of biomimetic 3D culture systems that replicate the molecular and cellular complexity of the bone marrow microenvironment [129,130,131]. The development of these approaches might help to overcome the limitations of primary JMML cultures and provide new physiologically relevant in vitro models to study the oncogenic mechanism driving JMML pathogenesis and resistance to therapy.

### 5.2. State-of-the-Art Methods to Explore JMML Tumor Origin, Heterogeneity and Evolution

Although the phenotype of JMML patients is dominated by the expansion of myeloid cells, there is evidence that also indicates the involvement of other hematopoietic lineages [4,20,132]. For this reason, JMML is considered a disease of the HSPC compartment [1,84,133]. However, very little is known about the cell hierarchies involved in leukemia progression, the intratumor heterogeneity, the specific identity of the leukemia initiating cells, or the clonal evolution of the disease. However, all these features might have an important impact in the prognosis of the disease and risk of relapse after HSCT. 

Over the last years, the rapid development of new technologies based on the use of next generation sequencing has changed the paradigms of cancer research. More specifically, the analysis of genomic, epigenomic, and/or transcriptomic information at single-cell resolution, and the comprehensive integration of these data can provide critical information on tumor origin, progression, and cellular heterogeneity.

In JMML, these approaches have only been applied to the transcriptomic analysis of HSPCs from two JMML patient samples by single-cell RNA sequencing (scRNA-seq) [134]. In this study, different JMML-specific HSPC clusters were identified, each of them showing upregulation of particular sets of genes, including myeloid genes, stem cell, and fetal genes, or genes associated to proliferation, leukemia, and erythroid differentiation [134]. These results demonstrated a broad heterogeneity within the JMML HSPC compartment, with the expression of aberrant transcriptional signatures that are not found in control cord blood HSPCs [134]. However, the collection of only transcriptional data and the low number of samples analyzed, limited the conclusions that could be extracted regarding the role of each of these clusters in tumor progression or maintenance.

Although not yet applied to JMML research, single-cell DNA-sequencing (scDNA-seq) combined with scRNA-seq has been successfully applied to the analysis of different types of cancers [135,136]. In these analyses, scRNA-seq data are used to identify specific cell clusters based on their transcriptomic profile. Then, this information is integrated with the scDNA-seq results to correlate different cell subpopulations with a specific mutational status and assess tumor clonal evolution during the differentiation process. In addition, transcriptomic data can be used to infer cell lineage differentiation between the annotated cell subpopulations by trajectory-based differential expression analysis using pseudotime analytical tools [137,138,139]. These bioinformatic approaches could be applied to the study of JMML tumoral heterogeneity and clonal evolution by tracking “first hit” mutations to a cell of origin, and studying their differentiation trajectories and acquisition of mutations. 

Similarly, JMML epigenomic intratumoral heterogeneity could be studied using methods that allow the analysis of DNA methylation at single-cell level. Simultaneous profiling of the transcriptome and DNA methylome from individual cells (scMT-seq) provides both a functional annotation of different cell subsets and a methylation profile [140]. A similar approach integrating scRNA-seq with methylome microarray data has been recently used to track the cell of origin in chronic lymphocytic leukemia [141]. The application of these technologies to the study of JMML could be critical to decipher the molecular mechanisms driving JMML leukemogenesis and progression.

## 6. Conclusions

The characterization of the genetic and epigenetic landscapes of JMML has significantly improved our understanding of the oncogenic pathways controlling the pathogenesis of this disease. Moreover, these analyses have turned the spotlight on DNA hypermethylation and secondary mutations as critical alterations that cooperate with canonical RAS pathway mutations and have an important prognostic value in the clinic. However, key questions regarding JMML origin, tumor cell identity, and intratumor and interpatient heterogeneity, remain open and must be addressed in the future. The use of new technologies allowing single-cell molecular profiling will be instrumental to achieve this aim and provide new relevant information on JMML pathobiology. This will in turn improve JMML diagnostic and prognostic criteria and contribute to identify potential therapeutic targets for the treatment of this disease.

## Figures and Tables

**Table 1:** Recurrent genetic alterations in JMML.

Pathway		Affected Gene	Alteration	%	References
RAS pathway	Tyrosine phosphatases	*PTPN11*	GoF-M	39%	[12,25,37,82]
	Tyrosine kinases	*ALK*	GoF-F	<3%	[37]
		*PDGFRB*	GoF-F	<3%	[65,66]
	RAS signaling components	*KRAS*	GoF-M	16%	[37,82]
		*NRAS*	GoF-M	15%	[37,82]
		*RRAS*	GoF-M	<3%	[29,64,83,84]
		*RRAS2*	GoF-M	<3%	[37,64]
	RAS regulators	*NF1*	LoF-M	11%	[37,57,82]
		*CBL*	LoF-M	11%	[38]
		*SOS1*	MS	<3%	[37]
JAK/STAT pathway		*SETBP1*	GoF-M	30%	[37,73,81,84,85]
		*JAK3*	GoF-M	8%	[29,73,74]
		*SH2B3*	LoF-M	7%	[64]
Hematopoietic commitment transcription factors		*RUNX1*	LoF-M	<3%	[64,84]
		*GATA2*	MS	<3%	[64]
Spliceosome components		*ZRSR2*	LoF-M	<3%	[29,64]
Epigenetic machinery	Histone modifiers	*DNMT3A*	LoF-M	3%	[64]
	PRC2 complex components and associated factors	*ASXL1*	LoF-M	8%	[64,84]
		*EZH2*	LoF-M	4%	[64]
		*SUZ12*	LOH	<3%	[29]
		*CDYL*	LOH	<3%	[29]

Abbreviations: GoF-M, gain-of-function mutations; GoF-F, gain-of-function gene fusions; LoF-M, loss-of-function mutations; MS, missense mutations (undetermined effect); LOH, loss of heterozygosity.

**Table 2:** Recurrent epigenetic alterations in JMML.

	Affected Gene	Alteration	%	References
Polypeptide from TGF-β superfamily of proteins	*BMP4*	Hypermethylation	36%	[94]
Family of G-protein-coupled receptors	*CALCA*	Hypermethylation	54%	[94]
Cyclin-dependent kinase inhibitor	*CDKN2B*	Hypermethylation	22%	[94]
Retinoic acid receptor	*RARB*	Hypermethylation	13%	[94]
RAS Regulator	*RASA4*	Hypermethylation	51%	[96]
Histone acetylation	*CREBBP*	Hypermethylation	77%	[97]
Scaffold protein in signal transduction	*AKAP12*	Hypermethylation	42%	[98]
